# Association Between Frailty and Cognitive Impairment in Chronic Kidney Disease: A Systematic Review and Meta‐Analysis

**DOI:** 10.1111/jnu.70110

**Published:** 2026-06-30

**Authors:** Hien Thi Bui, Renny Wulan Apriliyasari, Quyen Huong Quynh Bui, Pei‐Shan Tsai

**Affiliations:** ^1^ School of Nursing, College of Nursing Taipei Medical University Taipei Taiwan; ^2^ College of Health Sciences VinUniversity Hanoi Vietnam; ^3^ Department of Nursing Institut Teknologi Kesehatan Cendekia Utama Kudus Kudus Indonesia; ^4^ Faculty of Nursing and Medical Technology Can Tho University of Medicine and Pharmacy Can Tho Vietnam; ^5^ Department of Nursing and Research Center in Nursing Clinical Practice Wan Fang Hospital, Taipei Medical University Taipei Taiwan; ^6^ Research Center of Sleep Medicine Taipei Medical University Hospital Taipei Taiwan

## Abstract

**Background:**

The literature has reported conflicting findings regarding the association between frailty and cognitive impairment in patients with chronic kidney disease (CKD). This systematic review and meta‐analysis examined the association between frailty and cognitive impairment in patients with CKD.

**Methods:**

A comprehensive search of Embase, PubMed, Scopus, Web of Science, CINAHL, and the Cochrane Central Register of Controlled Trials was conducted from database inception to February 2026. Eligible studies comprised cross‐sectional, cohort, case–control, randomized controlled trials, and quasi‐experimental studies that reported associations between frailty and cognitive impairment in patients with CKD, with no time or language restrictions.

**Results:**

A total of 17 studies (9 cross‐sectional and 8 cohort) involving 129,868 patients with CKD (mean age 60.88 years) were included in the meta‐analysis. The findings indicated that patients with frailty with CKD had significantly higher odds of cognitive impairment than robust patients (odds ratio = 3.13, 95% CI 1.92–5.12). Heterogeneity in association size was influenced by study region, frailty measurement methods, and whether covariate adjustments were made during data analysis (all *p* < 0.05).

**Conclusions:**

This systematic review and meta‐analysis identified a significant association between frailty and cognitive impairment in patients with CKD. Future prospective cohort studies should assess the causal relationships between frailty and cognitive impairment in this population.

**Clinical Relevance Statement:**

This study systematically assessed the association between frailty and cognitive impairment in patients with CKD, addressing inconsistent findings in prior research. The results highlight the importance of implementing targeted interventions, particularly for nonfrail CKD patients, to reduce the burden of cognitive impairment and improve patients' outcomes.

## 1 Introduction

Chronic kidney disease (CKD) is a major global health problem that results from disease pathways related to diabetes, hypertension, obesity, and aging (Lv and Zhang 2019). One study conducted across 167 countries of individuals representing 97.4% of the global population reported a CKD prevalence of 9.5% (Bello et al. 2023). Frailty is a common condition among older populations and is a frequent comorbidity of CKD (Hoogendijk et al. 2019). A study reported that among patients with CKD, the prevalence of frailty is 38.1%, and the prevalence of prefrailty is 37.9% (Li et al. 2024). Although frailty considerably affects the quality of life and outcomes of patients with CKD, studies have suggested that this condition is dynamic and can be reversed with appropriate interventions (Lorenz et al. 2023; Mei et al. 2021; Nixon et al. 2020).

Cognitive impairment frequently cooccurs with CKD. One study revealed the prevalence of cognitive impairment among individuals with CKD to be 40% (Zhang et al. 2024). Additionally, the prevalence of cognitive dysfunction increases in individuals with advanced stages of CKD (Tamura et al. 2008). The severity of cognitive dysfunction in individuals with CKD ranges from mild to severe, and severe cases, such as dementia, substantially affect daily living and independence (Woodbridge et al. 2018). Patients with CKD who experience cognitive decline often have low health literacy and poor medication adherence, leading to insufficient self‐management and high mortality rates (Chan et al. 2022; Raphael et al. 2012).

The degree of patient frailty (robust, prefrail, or frail) is inversely associated with cognitive functions (Vahedi et al. 2022). Some systematic reviews and meta‐analyses have identified an association between incident frailty and cognitive dysfunction and reported adverse effects of frailty severity on cognitive function among older adults (Borges et al. 2019; Vahedi et al. 2022). However, these results may not be directly applicable to patients with CKD because of the unique association between decreased kidney function and cognitive decline and frailty. Because of the high prevalence of cognitive impairment and frailty in patients with CKD (Li et al. 2024; Zhang et al. 2024), exploring the association between these conditions is crucial to developing appropriate treatment strategies.

Evidence from a prior systematic review indicates that CKD is associated with increased risk of both frailty and cognitive impairment (Shen et al. 2017). The review further suggests that these conditions may arise from shared underlying pathophysiological mechanisms that encompass both vascular and nonvascular pathways in patients with CKD. Additionally, several observational studies have reported a higher prevalence of cognitive dysfunction among individuals with prefrailty and frailty than among physically robust individuals (Lee et al. 2021; McAdams‐DeMarco et al. 2015; Vettoretti et al. 2020). However, not all studies have reported an association between frailty and cognitive dysfunction (Franco et al. 2023; Guo et al. 2022). One systematic review and meta‐analysis demonstrated that cognitive function was significantly associated with frailty in patients with CKD (Li et al. 2024); however, that study's pooled findings were derived from only 2 studies published before 2020. Notably, both studies in that meta‐analysis used the Mini‐Mental State Examination (MMSE), an instrument with well‐reported limitations, including the possibility of missing mild cognitive impairment or generating false positives (Devenney and Hodges 2017). Consequently, such misclassification of cognitive status could bias pooled effect sizes toward an association between frailty and cognitive impairment. Since 2020, several studies have reported an association between frailty and cognitive function using a variety of standardized and well‐validated instruments such as the Montreal Cognitive Assessment (MoCA) and Modified Mini‐Mental State Examination (3MS) to improve this estimation (Anderson et al. 2023; Chu et al. 2021; Guo et al. 2022).

The coexistence of frailty and cognitive impairment is linked to poor outcomes, such as an increased risk of all‐cause mortality and dementia (Bu et al. 2021; Zheng et al. 2020). Hence, the association between frailty and cognitive dysfunction in patients with CKD must be validated by pooling results from studies that have used different frailty and cognitive assessment tools. In consideration of this, the present study examined the association between frailty and cognitive impairment among patients with CKD.

## 2 Design and Method

This systematic review was conducted and reported following the Preferred Reporting Items for Systematic Reviews and Meta‐Analyses statement (Page et al. 2021). The review was registered with PROSPERO (CRD42025630949).

### 2.1 Search Strategy and Selection Criteria

We conducted a comprehensive search of Embase, PubMed, Scopus, Web of Science, CINAHL, and the Cochrane Central Register of Controlled Trials from the inception date of each database to February 2026, with no restrictions imposed on time and language. The terms “frailty,” “cognitive impairment,” and “chronic kidney disease,” along with their synonyms, were searched in the aforementioned databases in Keywords, Titles, and Abstracts; we also searched for these terms in Medical Subject Headings and Emtree (Table S1). Finally, we manually searched the reference lists of the included studies to identify additional potentially eligible studies.

Studies that (1) used a cross‐sectional, cohort, case–control, randomized control trial (RCT), or quasi‐experimental study design; (2) included participants who had received a diagnosis of CKD at the age of 18 years or older; (3) reported odds ratios (ORs) and 95% CIs or data that could be used to calculate ORs and CIs for assessing the association between frailty and cognitive impairment; and (4) assessed frailty and cognitive impairment using recognized and validated instruments were included. Reviews, case reports, letters, commentaries, and conference abstracts were excluded.

### 2.2 Study Selection and Data Extraction

We used EndNote software for the study selection process. After removal of duplicate papers, two researchers independently assessed the titles, abstracts, and full texts. The inconsistent opinions would be discussed or consulted with the third reviewer.

Two researchers independently extracted data. Any disagreement was resolved through discussion with a third researcher. The extracted data comprised the characteristics of the included studies, namely, the study authors, continent, design, duration of follow‐up for cohort studies, sample size, hemodialysis status, mean patient age (years), number of male patients (%), patient smoking status (% yes), patient BMI, patient time on hemodialysis (months), number of patients with diabetes (% yes), number of patients with hypertension (% yes), number of patients with stroke (% yes), and measurements and cutoff values of cognitive impairment and frailty (frailty measurements, cutoff values of frailty, cognitive impairment measurement, cutoff values of cognitive impairment, covariates adjusted, prevalence of frailty in patients with CKD and cognitive impairment, and prevalence of cognitive impairment in patients with CKD and frailty). If studies reported both unadjusted and adjusted values, we extracted the most comprehensive adjusted ORs to reduce confounding. For longitudinal studies, we extracted baseline data to ensure consistency in frailty and cognitive impairment values across comparisons. We also contacted the corresponding authors of the studies to obtain additional data to calculate ORs.

### 2.3 Assessment of Study Quality

Two independent researchers assessed the quality of the included studies by using the Newcastle‐Ottawa Scale for nonrandomized studies (Wells et al. 2000). The study quality was scored using a “star system,” with a point awarded for each criterion met. In cohort and case–control studies, quality was assessed across 3 domains: selection (4 points), comparability (2 points), and outcomes/exposure (3 points). The 8‐item instrument used for quality assessment rated studies as low risk of bias (8–9 points), medium risk of bias (6–7 points), and high risk of bias (≤ 5 points). Selection, comparability, and outcomes were assessed for cross‐sectional studies. Studies with a score of 7 to 8 points were classified as having a low risk of bias, those with a score of 6 points were classified as having a medium risk of bias, and those with a score of ≤ 5 points were classified as having a high risk of bias. Additionally, the revised Cochrane Risk of Bias tool was pre‐specified for assessing the methodological quality of RCTs, if any were identified. This instrument includes five domains and an overall bias judgment, categorized as low risk of bias, some concerns, or high risk of bias (Minozzi et al. 2022).

### 2.4 Statistical Analyses

Our data analysis was conducted using Comprehensive Meta‐Analysis software, version 2.0 (Biostat, Englewood, NJ, USA). Pooled ORs and prevalence were utilized to investigate the association between frailty and cognitive impairment, and the prevalence of frailty and cognitive impairment among patients with CKD by using random‐effect models. We used the Cochran *Q* and *I* ^2^ statistics to determine the proportion of total variation and evaluate heterogeneity across studies. The results were classified as indicating low, moderate, or high levels of heterogeneity on the basis of the *I* ^2^ cutoff points of 25%, 50%, and 75%, respectively (Higgins and Thompson 2002). A *p*‐value < 0.1 in *Q* statistics or an *I* ^2^ value of at least 50% indicated substantial heterogeneity. Meta‐regression and moderator analyses were conducted when heterogeneity was identified. For moderator analyses, subgroup differences were examined using mixed‐effect models, in which pooled effects within subgroups were estimated using random‐effects models and between‐group differences were tested using fixed‐effect comparisons. We used Begg's rank correlation and Egger's regression tests with the significance level set at *p* < 0.05 to evaluate publication bias. Several sensitivity analyses were performed to examine the robustness of the findings. First, the pooled adjusted estimate was calculated by excluding the study with the largest OR. Second, given the recognized limitations of the MMSE, particularly its lower sensitivity for mild cognitive impairment, an additional sensitivity analysis was performed by excluding studies that used the MMSE to assess cognitive impairment.

## 3 Results

**FIGURE 1:**
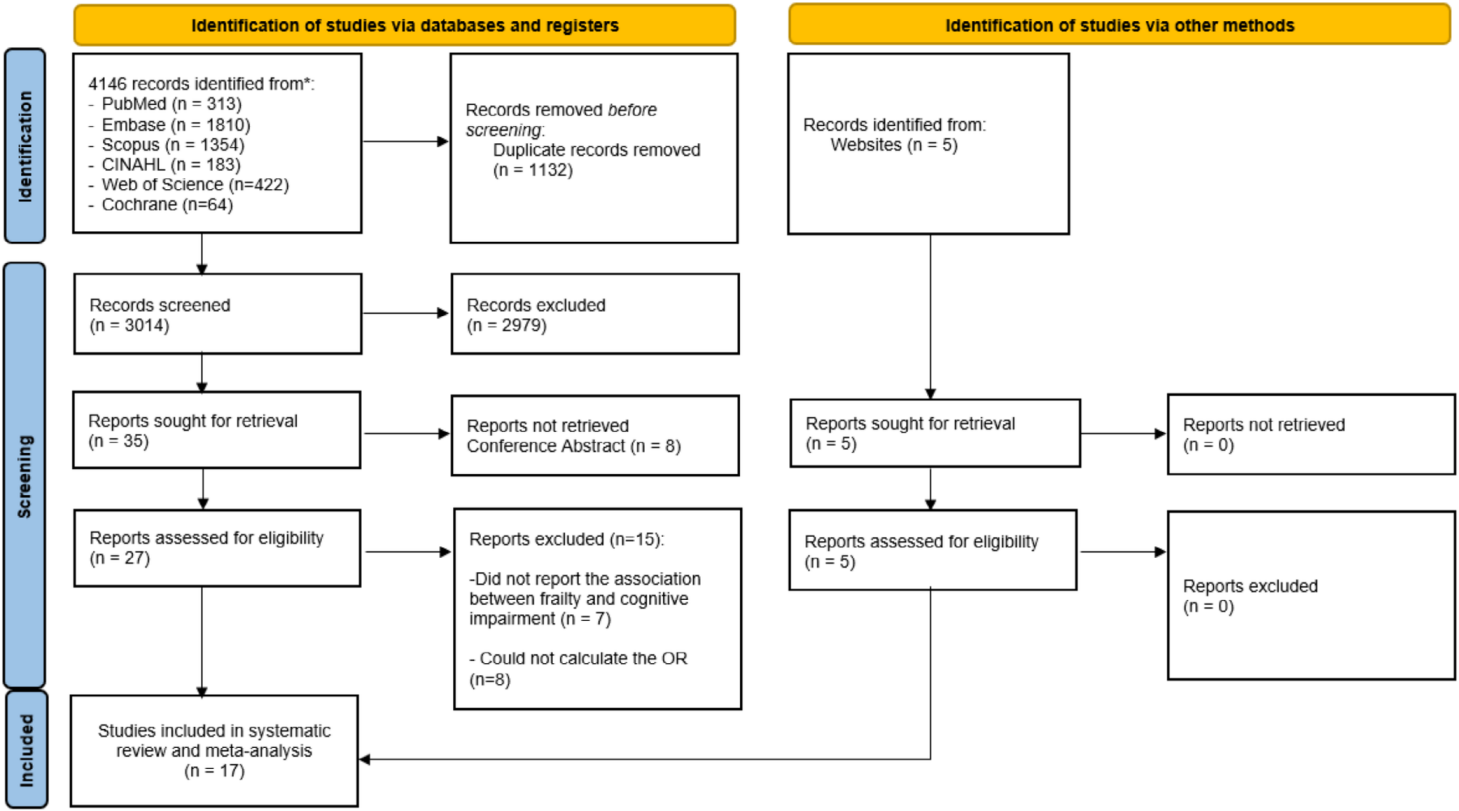
PRISMA 2020 flow diagram.

We initially identified 4146 articles. After exclusion of duplicates and articles that did not meet the inclusion criteria, 17 studies—9 cross‐sectional studies (Chen et al. 2025; Erken et al. 2019; Erken and Erken 2023; Gesualdo et al. 2020; Hong et al. 2024; Nixon et al. 2020; Novais et al. 2021; Poveda et al. 2016; Vettoretti et al. 2020) and 8 cohort studies (Anderson et al. 2023; Chu et al. 2021; Franco et al. 2023; Guo et al. 2022; Jafari et al. 2020; Lee et al. 2021; McAdams‐DeMarco et al. 2015; Thind et al. 2022)—were included in our systematic review and meta‐analysis (shown in Figure 1). Table S2 lists the studies excluded after the full‐text review.

### 3.1 Study Characteristics

The characteristics of the included studies are presented in Table 1. The 17 included studies involved 129,868 participants with a mean age of 60.88 years. In total, 5, 5, and 7 studies were conducted in the Americas (Chu et al. 2021; Gesualdo et al. 2020; Hong et al. 2024; Jafari et al. 2020; McAdams‐DeMarco et al. 2015), Asia (Chen et al. 2025; Erken et al. 2019; Erken and Erken 2023; Guo et al. 2022; Lee et al. 2021), and Europe (Anderson et al. 2023; Franco et al. 2023; Nixon et al. 2020; Novais et al. 2021; Poveda et al. 2016; Thind et al. 2022; Vettoretti et al. 2020), respectively. While almost all studies were reported in English, one study was presented in Turkish (Erken et al. 2019). The percentage of men was 57.12%, and 1.43% of the participants had previously smoked.

**TABLE 1:** Characteristics of included studies (*n* = 17).

Author(s), year	Continent	Study design	Duration of follow‐up (for cohort study)	Sample size	Hemodialysis status	Mean age (years)	Men (%)	Smoking status (% yes)	BMI	Time on hemodia‐lysis (months)	Diabetes (% Yes)	Hyper‐tension (% Yes)	Stroke (% Yes)
Anderson et al. (2023)	Europe	Prospective cohort study	Beyond 1 year	448	Under hemodialysis	63.49	57.37	14.96	27.28	43.43	29.69	N/I	12.28
Chu et al. (2021)	America	Prospective cohort study	Johns Hopkins Hospital: 10 years University of Michigan Medical Center: 22 months	3854	Under hemodialysis	55.00	59.40	N/I	N/I	14.28	N/I	N/I	N/I
Erken and Erken (2023)	Asia	Cross‐sectional study	N/I	156	Nonhemodialysis	69.11	48.10	N/1	29.19	N/I	N/I	N/I	N/I
Erken et al. (2019)	Asia	Cross‐sectional study	N/I	102	Under hemodialysis	48.33	64.70	N/I	24.70	64.66	N/I	N/I	N/I
Franco et al. (2023)	Europe	Prospective cohort study	2 years	100	Nonhemodialysis	78.80	62.00	N/1	27.84	N/I	46.81	92.55	13.83
Gesualdo et al. (2020)	America	Cross‐sectional study	N/I	107	Under hemodialysis	54.30	67.30	N/I	N/I	48.91	N/I	N/I	N/I
Guo et al. (2022)	Asia	Prospective cohort study	1 year	204	Under hemodialysis	71.65	55.40	46.60	23.95	59.00	41.20	86.80	15.20
Hong et al. (2024)	America	Cross‐sectional study	N/I	1161	Both conditions	N/I	43.40	52.10	N/I	N/I	33.40	86.20	12.50
Jafari et al. (2020)	America	Prospective cohort study	1 year	97	Under hemodialysis	62.86	58.00	N/I	29.33	35.50	51.20	79.60	N/I
Lee et al. (2021)	Asia	Prospective cohort study	6 years	122,407	Both conditions	61.00	44.20	0.70	N/I	N/I	N/I	70.50	N/I
McAdams‐DeMarco et al. (2015)	America	Prospective cohort study	1 year	324	Under hemodialysis	54.80	56.50	23.13	29.71	N/I	56.17	100	N/I
Nixon et al. (2020)	Europe	Cross‐sectional study	N/I	90	N/I	69.00	50.00	54.00	29.00	N/I	27.00	N/I	N/I
Novais et al. (2021)	Europe	Cross‐sectional study	N/I	90	Both conditions	74.20	69.20	N/1	26.90	N/I	44.90	91.00	10.90
Poveda et al. (2016)	Europe	Cross‐sectional study	N/I	83	Under hemodialysis	65.92	53.01	N/I	25.72	44.77	34.94	56.63	N/I
S. Vettoretti et al. (2020)	Europe	Cross‐sectional study	N/I	112	Nonhemodialysis	80.00	70.00	N/I	28.00	N/I	N/I	N/I	N/I
Thind et al. (2022)	Europe	Prospective cohort study	N/I	208	Under hemodialysis	65.00	65.90	N/I	N/I	N/I	N/I	N/I	N/I
Chen et al. (2025)	Asia	Cross‐sectional study	N/I	325	Under hemodialysis	61.00	66.80	12.90	22.25	N/I	62.80	N/I	N/I

Abbreviations: BMI, body mass index; CKD, chronic kidney disease; N/I, no information.

Ten studies were conducted among patients receiving hemodialysis (Anderson et al. 2023; Chen et al. 2025; Erken et al. 2019; Gesualdo et al. 2020; Guo et al. 2022; Jafari et al. 2020; McAdams‐DeMarco et al. 2015; Poveda et al. 2016; Chu et al. 2021; Thind et al. 2022), with the average time on hemodialysis being 21.6 months. In total, 40.42%, 70.84%, and 12.72% of the patients had a history of diabetes, hypertension, and stroke, respectively.

Regarding frailty measurements (Table S3), most studies (*n* = 12) evaluated frailty using the Fried Frailty Phenotype (Anderson et al. 2023; Chen et al. 2025; Chu et al. 2021; Franco et al. 2023; Gesualdo et al. 2020; Guo et al. 2022; Hong et al. 2024; Jafari et al. 2020; McAdams‐DeMarco et al. 2015; Nixon et al. 2020; Novais et al. 2021; Vettoretti et al. 2020), followed by the Clinical Frailty Index (*n* = 2) (Erken et al. 2019; Erken and Erken 2023); the Edmonton Frailty Scale (*n* = 1) (Thind et al. 2022); the fatigue, resistance, ambulation, illness, and loss of weight (FRAIL) questionnaire (*n* = 1) (Poveda et al. 2016); and the modified FRAIL scale (*n* = 1) (Lee et al. 2021). In studies applying the Fried Frailty Phenotype, participants who met at least 3 criteria were categorized as frail, whereas those meeting 1 or 2 criteria were considered as prefrail (Chen et al. 2025; Gesualdo et al. 2020; Guo et al. 2022; Jafari et al. 2020; Novais et al. 2021; Poveda et al. 2016).

To assess cognitive impairment, 7 studies used MoCA (Anderson et al. 2023; Erken et al. 2019; Erken and Erken 2023; Franco et al. 2023; Guo et al. 2022; Jafari et al. 2020; Thind et al. 2022), 5 utilized the MMSE (Chen et al. 2025; Nixon et al. 2020; Novais et al. 2021; Poveda et al. 2016; Vettoretti et al. 2020), 2 applied 3MS (Chu et al. 2021; McAdams‐DeMarco et al. 2015), one used Addenbrooke's Cognitive Examination‐Revised (ACE‐R) (Gesualdo et al. 2020), one used several objective measures to assess global and domain‐specific cognitive functions (Hong et al. 2024), and one used a validated algorithm to assess clinical cognitive impairment (Lee et al. 2021). Potential covariates were adjusted in the statistical analyses of the studies of Anderson et al. (2023) and Chu et al. (2021).

### 3.2 Quality of Included Studies

Since all included studies were observational, the quality assessment was performed by the use of the Newcastle‐Ottawa Scale (Table S4). The results showed that most included studies exhibited medium risk (*n* = 7) (Chu et al. 2021; Erken et al. 2019; Erken and Erken 2023; Gesualdo et al. 2020; Jafari et al. 2020; Nixon et al. 2020; Poveda et al. 2016); 7 exhibited low risk (Anderson et al. 2023; Chen et al. 2025; Franco et al. 2023; Guo et al. 2022; Hong et al. 2024; Lee et al. 2021; McAdams‐DeMarco et al. 2015), and 3 exhibited high risk (Novais et al. 2021; Thind et al. 2022; Vettoretti et al. 2020).

### 3.3 Narrative Review of the Association Between Frailty and Cognitive Impairment in Cohort Studies

Among the 8 cohort studies in this review, 2 reported on the association between frailty and cognitive function over time, that is, at baseline and at a 1‐year follow‐up (Jafari et al. 2020; McAdams‐DeMarco et al. 2015). Their findings indicated that frailty was independently associated with reduced cognitive function, as determined by 3MS scores at 1‐year follow‐up, with a significant dose–response in levels of frailty (−2.8 points; 95% CI −5.4 to −0.2; *p* = 0.03) (McAdams‐DeMarco et al. 2015). Additionally, Jafari et al. (2020) used the MoCA questionnaire and uncovered a significant difference in cognitive impairment between frail (Median = 24, interquartile range [IQR] = 19–25) and nonfrail (Median = 25, IQR = 21–26) groups at a 1‐year follow‐up (*p* = −0.04). Moreover, Lee et al. (2021) reported that individuals with diabetic kidney disease with frailty had a higher risk of developing cognitive impairment after 3.68 years of follow‐up, after demographic and lifestyle factors, medication, comorbidities, and interventions were controlled for.

### 3.4 Meta‐Analysis Results

#### 3.4.1 Association Between Frailty and Cognitive Impairment

**FIGURE 2:**
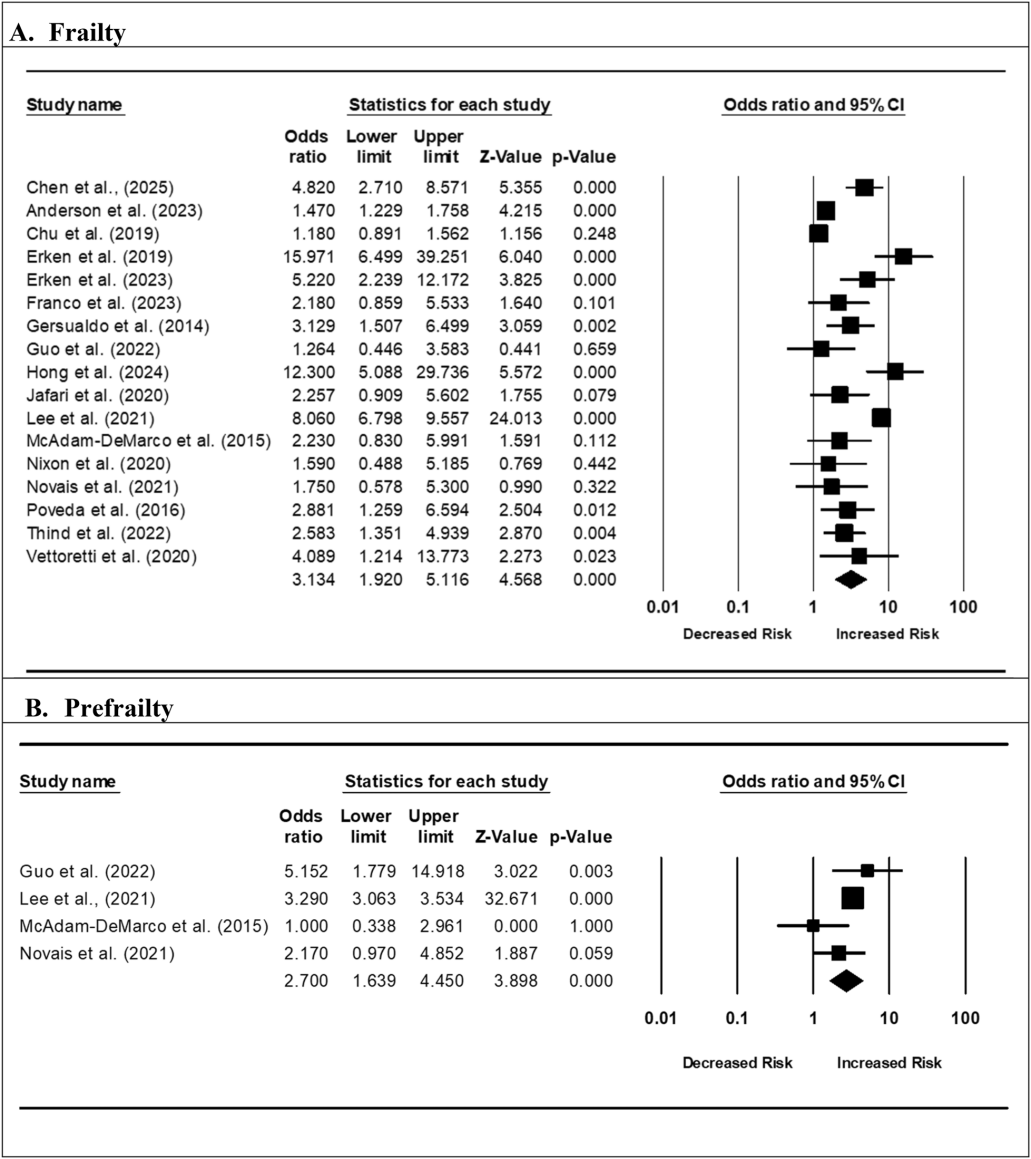
(A) Odds of cognitive impairment in frail patients with chronic kidney disease compared with robust patients. (B) Odds of cognitive impairment in prefrail patients with chronic kidney disease compared with robust patients.

Figure 2 illustrates the association between frailty (frailty vs. nonfrailty) and cognitive impairment among CKD patients (*n* = 17). The pooled OR was 3.13 (95% CI 1.92–5.12), suggesting that frail CKD patients had a 213% increase in the likelihood of cognitive impairment than robust CKD patients. The values of *Q* and *I* ^2^ indicated substantial heterogeneity among the included studies (*Q* = 265.96, *p* < 0.001; *I* ^2^ = 93.98%).

The patients with CKD with prefrailty had a greater likelihood of experiencing cognitive impairment (*n* = 4, pooled OR = 2.70, 95% CI 1.64–4.45) than those without prefrailty did. Heterogeneity in these studies was unlikely (*Q* = 6.31, *p* = 0.10, *I* ^2^ = 52.42%).

#### 3.4.2 Moderator, Meta‐Regression, and Sensitivity Analyses

As shown in Table 2, the region in which the studies were conducted, the type of frailty measurements used, and whether the study employed covariate adjustments were significant sources of heterogeneity (*p* = 0.01, *p* = 0.02, and *p* < 0.001, respectively). The pooled OR for the association between frailty and cognitive impairment was significantly higher in the studies conducted in Asia than in those conducted in the other continents (pooled ORs = 5.67 vs. 2.32). Additionally, in the studies that utilized the Fried Frailty Phenotype to assess frailty, the patients had significantly lower odds of cognitive impairment than the patients in the studies that used other tools such as the Clinical Frailty Index, Edmonton Frailty Scale, FRAIL questionnaire, or modified FRAIL scale did (pooled ORs = 2.38 vs. 5.50). Moreover, the studies that adjusted covariates in assessing the association between frailty and cognitive impairment reported lower odds of patient cognitive impairment than those that did not did (pooled ORs = 1.35 vs. 3.69). Substantial heterogeneity persisted within subgroups, even after conducting moderator analyses. In contrast, our findings suggested that the mean age, gender, BMI, time on hemodialysis, the percentages of diabetes, hypertension, stroke, and smoking status as well as cognitive impairment measurement and MoCA cut‐off value, were not sources of heterogeneity (all *p*‐value > 0.05).

**TABLE 2:** Moderator analysis and meta‐regression for the odds of cognitive impairment among patients with chronic kidney disease with frailty.

Sample characteristics				
Meta regression	*n*	*β* coefficients	95% CI	*p*
Mean age	16	−0.02	−0.07–0.02	0.29
Percentage of men	17	−0.02	−0.07–0.02	0.32
BMI	12	−0.09	−0.27–0.09	0.32
Time on hemodialysis	7	0.03	−0.001–0.06	0.06
Percentage of diabetes	10	0.01	−0.03–0.05	0.59
Percentage of hypertension	8	−0.02	−0.06–0.03	0.47
Percentage of stroke	5	−0.12	−0.75–0.51	0.72
Percentage of smoking history	**6**	−0.002	−0.05–0.04	0.94

Subgroup analysis	*n*	Pooled OR	95% CI	*I* ^2^	*p*
**Region**					
Asia	5	5.67	3.19–10.10	77.26	0.01
Other continents	12	2.32	1.66–3.24	70.10	
**Cognitive impairment measurement**					
MoCA	7	2.88	1.56–5.35	83.12	0.78
Others	10	3.29	1.66–6.54	93.88	
**MoCA cut‐off value**					
26	4	2.16	1.21–3.86	71.82	0.34
Below 26	3	4.30	1.17–15.76	83.54	
**Frailty measurement**					
Fried Frailty Phenotype	12	2.38	1.64–3.46	76.49	0.02
Others	5	5.50	3.01–10.05	79.52	
**Covariate adjustment**					
No	15	3.69	2.49–5.48	77.86	< 0.001
Yes	2	1.35	1.10–1.67	40.25	

Abbreviations: BMI, Body Mass Index; MoCA, Montreal Cognitive Assessment.

The sensitivity analysis confirmed the robustness of the findings (Table S5). After removing the study with the largest OR, the results remained statistically significant (OR = 2.84, 95% CI 1.73–4.68), indicating that the odds of cognitive impairment were higher in patients with CKD and frailty than in those without frailty. Similarly, after excluding the five studies that assessed cognitive function using the MMSE, the pooled estimate remained statistically significant (OR = 3.26, 95% CI 1.78–5.98), further supporting a robust association between frailty and cognitive impairment in patients with CKD.

#### 3.4.3 Prevalence of Frailty Among Patients With CKD With Cognitive Impairment

**FIGURE 3:**
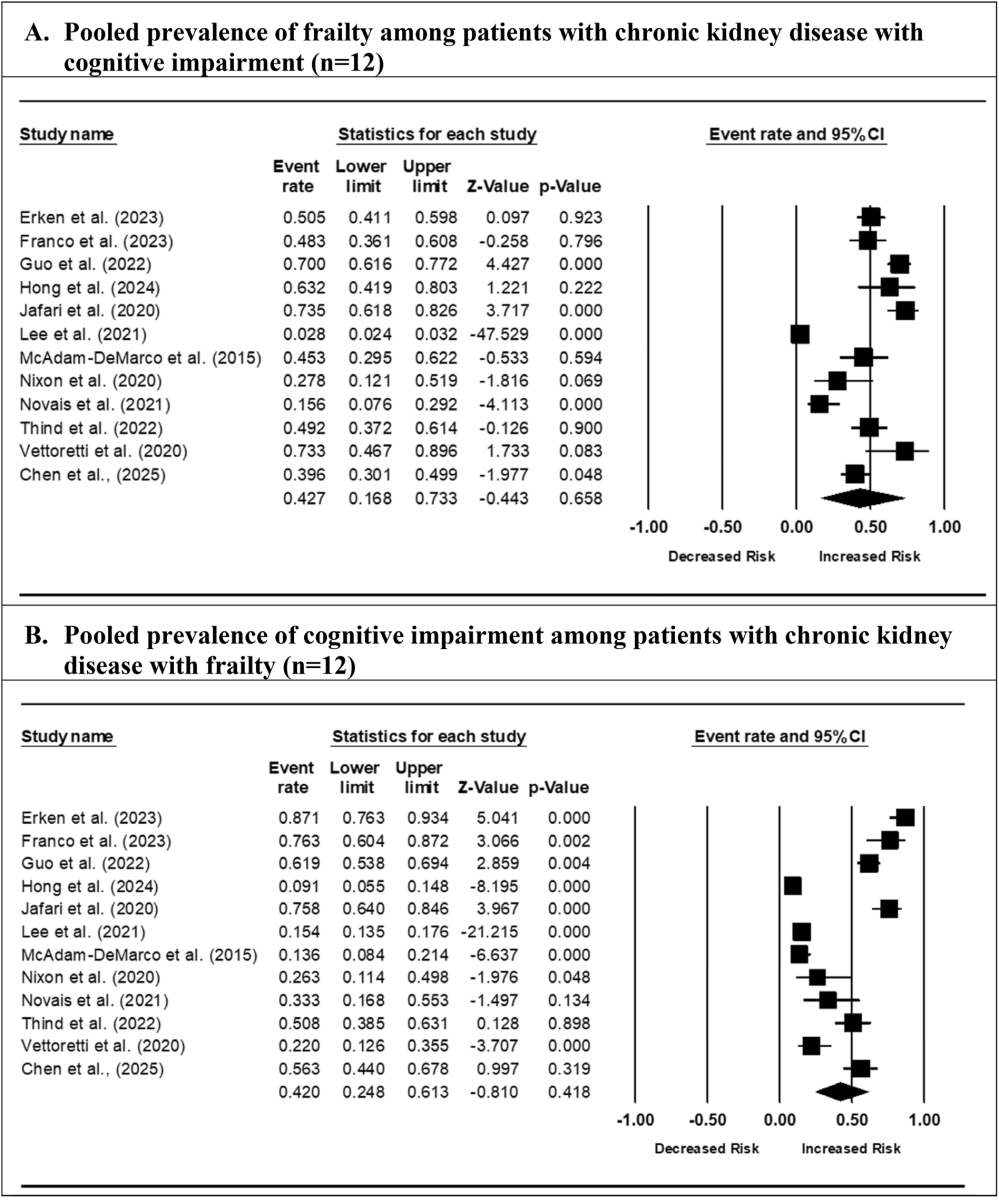
(A) Pooled prevalence of frailty among patients with chronic kidney disease with cognitive impairment. (B) Pooled prevalence of cognitive impairment among patients with chronic kidney disease with frailty.

The pooled prevalence of frailty among CKD patients with cognitive impairment was 42.70% in a total sample of 7238 cases of CKD patients with cognitive impairment, shown in Figure 3 (*n* = 12, 95% CI 0.17–0.73), with a high level of heterogeneity (*Q* = 1142.18, *p* < 0.001, *I* ^2^ = 99.04%).

#### 3.4.4 Prevalence of Cognitive Impairment Among Patients With CKD and Frailty

The included studies involved 2010 cases of frailty (*n* = 12). The overall prevalence of cognitive impairment among CKD patients and frailty was 42% (*Q* = 372.08, *p* < 0.001, *I* ^2^ = 97.04%).

#### 3.4.5 Publication Bias

The findings indicate that publication bias was unlikely to be present in the included studies (Begg test: *p* = 0.43, and Egger test: *p* = 0.95).

## 4 Discussion

This meta‐analysis of seventeen studies revealed that the patients with CKD who acquired frailty were associated with a 213% increase in odds of cognitive impairment than those who did not. Additionally, patients with CKD who had suffered from prefrailty were associated with 170% higher odds of impairment than those without frailty.

Our findings support the argument that frailty is a substantial risk factor for cognitive impairment among patients with CKD. One systematic review concluded that frailty was a predictor of cognitive disorders, particularly vascular dementia, among older adults on the basis of the pooled results from 6 cohort studies (Borges et al. 2019). Although the results from our meta‐analysis mostly provided cross‐sectional data to support the association between frailty and cognitive impairment among patients with CKD, several cohort studies in the present narrative review reported that frailty was significantly associated with cognitive dysfunction in individuals with CKD (Lee et al. 2021; McAdams‐DeMarco et al. 2015). A 3.68‐year longitudinal study among 149,145 patients with diabetic kidney disease revealed that patients who reported 1, 2, and > 2 FRAIL scale items had a significantly greater risk of cognitive impairment than their physically robust counterparts did (Lee et al. 2021). Therefore, frailty may be a modifiable risk factor for preventing cognitive impairment in patients with CKD.

The findings from the present meta‐analysis also revealed both the prevalence of frailty among patients with CKD with cognitive impairment and that of cognitive impairment among patients with CKD with frailty are sizable (both > 40%). Consistent with our observations, an earlier study involving 761 older adults without baseline cognitive impairment reported that frailty increased the risk of developing mild cognitive impairment by 63%, even after adjustment for confounding factors such as cardiovascular diseases, depressive symptoms, disability, and vascular risk factors (Boyle et al. 2010). Additionally, in another meta‐analysis, individuals with frailty tended to score lower on the MMSE than physically robust individuals did, and patients with CKD with prefrailty had a higher risk of cognitive impairment than those without frailty did (Vahedi et al. 2022). Moreover, a study involving 122 older adults with cognitive impairment demonstrated that cognitive status (assessed using longitudinal Clinical Dementia Rating‐Sum of Boxes scores) and age were significantly associated with longitudinal frailty scores (Chong et al. 2015). These findings suggest that frailty and cognitive impairment likely exhibit a bidirectional relationship (de Morais Fabrício et al. 2020; Halil et al. 2015; Robertson et al. 2013).

Studies have also suggested that frailty and cognitive impairment may have similar pathophysiological mechanisms, although the precise pathways remain unclear (Robertson et al. 2013; Sargent et al. 2018). The conditions have common risk factors, such as chronic illnesses, poor cardiovascular health, inflammation, or hormonal imbalances. Neuropathological changes may directly affect frailty indicators, potentially contributing to cognitive decline (Robertson et al. 2013). Hence, exploring the connection between frailty and cognitive impairment is crucial in managing the condition of patients with CKD and cognitive impairment. Because frailty often leads to disability and a loss of independence (Kojima 2017), such patients should be proactively assessed for frailty.

Our analysis uncovered an increased likelihood of cognitive impairment among patients with CKD with frailty; however, the results exhibited heterogeneity. Substantial sources of heterogeneity in the analysis were the region in which the studies were conducted, the type of frailty measurements used, and whether covariate adjustments were applied. In our moderator analysis, patients with frailty in Asia had higher odds of cognitive impairment than those in other continents. The results of another systematic review and meta‐analysis also revealed that in terms of region, the prevalence of cognitive impairment among patients with CKD was highest in Asia (44%), followed by the Americas (37%) and Europe (34%) (Zhang et al. 2024). The deficit accumulation model has similarly indicated that the prevalence of frailty among patients with CKD is highest in Asia (25%), followed by the Americas (23%) and Europe (22%) (O'Caoimh et al. 2021). Hence, our findings are consistent with broader population‐based studies regarding the prevalence of both conditions in individuals with CKD.

The studies included in our meta‐analysis utilized several tools to assess cognitive function, specifically, the MoCA, MMSE, 3MS, ACE‐R, and objective measures. Research has demonstrated that the MoCA is more sensitive than the MMSE is in detecting mild cognitive impairment (Nasreddine et al. 2005). Additionally, a smaller study involving 42 patients receiving hemodialysis and 42 healthy controls revealed that the MoCA outperformed the MMSE in neurocognitive assessments (Tiffin‐Richards et al. 2014). However, despite the use of various cognitive assessments, a significant association between frailty and cognitive impairment was consistently observed in the studies we reviewed. Neither the choice of cognitive impairment measurement nor the specific MoCA cutoff value explained the variations in the strength of this association. These results underscore the stable association between frailty and cognitive impairment across assessment tools. Nevertheless, the present study's results also revealed that studies using the Fried Frailty Phenotype reported a significantly reduced likelihood of cognitive impairment relative to that reported in those employing other frailty assessment tools such as the Clinical Frailty Index, Edmonton Frailty scale, FRAIL scale, and modified FRAIL scale. The Fried Frailty Phenotype remains the most commonly used tool for frailty assessment because it was specifically designed to identify frailty as a risk factor for adverse health outcomes among community‐dwelling older adults, making the discrepancy in the findings of the studies using this measurement versus others a concern that warrants further investigation (Buta et al. 2016).

Although the studies that applied covariate adjustments reported significantly lower odds ratios, both adjusted and unadjusted subgroups showed a significant association between frailty and cognitive impairment in patients with CKD. However, only 2 studies applied covariate adjustment, highlighting a gap in the literature and a need for research to assess the association between frailty and cognitive impairment while accounting for potential confounding factors. Such research may provide stronger evidence to confirm the association between these health conditions.

This study has several limitations. First, most included studies were cross‐sectional, and baseline data in cohort studies were used to evaluate the association between frailty and cognitive impairment, precluding the establishment of causal relationships between these health conditions. To address this limitation, future research should include prospective cohort studies and assess the causal link between frailty and cognitive impairment among individuals with CKD. Second, only 2 studies in this meta‐analysis accounted for confounding factors, which may have led to residual confounding. Future meta‐analyses should incorporate other adjusted OR values to provide a more accurate estimation of the association between frailty and cognitive impairment in individuals with CKD.

Because cognitive impairment is a key contributor to the burden of aging‐related CKD (Murray 2008), addressing such impairment is essential to reducing this burden. Targeted interventions, particularly for nonfrail individuals, may mitigate this burden and improve outcomes in this population.

## 5 Conclusion

This study highlights a significant association between frailty and cognitive impairment among the CKD population. The magnitude of association varies with the different tools used to assess frailty. Our findings evidence the importance of screening and managing frailty to prevent cognitive impairment in patients with CKD. Additionally, prospective cohort studies should be implemented to assess the causal relationship between frailty and cognitive impairment.

## Disclosure

Clinical resources: Frailty: aging with kidney problems: https://kidneycareuk.org/kidney‐disease‐information/living‐with‐kidney‐disease/patient‐info‐frailty‐ageing‐with‐kidney‐problems/#who‐can‐help‐me. Kidney disease, kidney transplant, and frailty: https://frailtyscience.org/clinical‐topics/nephrology/.

## Ethics Statement

This study is a systematic review and meta‐analysis of previously published data and did not require ethical approval. The review was registered with PROSPERO (Registration No. CRD42025630949).

## Conflicts of Interest

The authors declare no conflicts of interest.

## Supporting information


**Table S1:** Keywords from PubMed, Embase, Scopus, Web of Science, CINAHL, and Cochrane.
**Table S2:** A list of excluded studies after a full‐text review.
**Table S3:** Measurements and Cutoff Values of Cognitive Impairment and Frailty in Included Studies (*n* = 17).
**Table S4:** Quality Assessment of Included Studies (*n* = 17).
**Table S5:** Sensitivity analysis.

## Data Availability

The data that support the findings of this study are available on request from the corresponding author. The data are not publicly available due to privacy or ethical restrictions.
