# Supplementary material for: Association Between Frailty and Cognitive Impairment in Chronic Kidney Disease: A Systematic Review and Meta‐Analysis
Source: J Nurs Scholarsh. 2026 Jun 30;58(4):e70110. doi: 10.1111/jnu.70110 (PMC13316728; doi:10.1111/jnu.70110)
Supplement: Supplementary file 1 — Table S1: Keywords from PubMed, Embase, Scopus, Web of Science, CINAHL, and Cochrane. Table S2: A list of excluded studies after a full‐text review. Table S3: Measurements and Cutoff Values of Cognitive Impairment and Frailty in Included Studies (n = 17). Table S4: Quality Assessment of Included Studies (n = 17). Table S5: Sensitivity analysis. [file JNU-58-0-s001.docx]

**SUPPLEMENTARY MATERIAL**

**Association between Frailty and Cognitive Impairment in Chronic Kidney Disease: A Systematic Review and Meta-Analysis**

**Table of Contents**

[Supplementary Table S1 Keywords from PubMed, Embase, Scopus, Web of Science, CINAHL, and Cochrane 2](#_Toc233140979)

[Supplementary Table S2. A list of excluded studies after a full-text review 10](#_Toc233140980)

[Supplementary Table S3. Measurements and Cutoff Values of Cognitive Impairment and Frailty in Included Studies (n=17) 12](#_Toc233140981)

[Supplementary Table S4. Quality Assessment of Included Studies (n = 17) 14](#_Toc233140982)

[Supplementary Table S5. Sensitivity analysis 16](#_Toc233140983)

Supplementary Table S1 Keywords from PubMed, Embase, Scopus, Web of Science, CINAHL, and Cochrane

The most recent search was conducted on February 27, 2026.

**Summary Table of Databases Results**

| Database | Total |
| --- | --- |
| PubMed | 313 |
| Embase | 1810 |
| Scopus | 1354 |
| Web of Science | 422 |
| CINAHL | 183 |
| Cochrane | 64 |
| Total | 4146 |

|  | # | Search strategy |
| --- | --- | --- |
| **1. Pubmed**  **(n=313)** | 1 | frailty[Title/Abstract] OR frailty[MeSH Terms] |
|  | 2 | 'Frail*'[Title/Abstract] |
|  | 3 | 'Pre-frail'[Title/Abstract] |
|  | 4 | 'Prefrail'[Title/Abstract] |
|  | 5 | Debility[Title/Abstract] |
|  | 6 | 'Debilit*'[Title/Abstract] |
|  | 7 | 'Frailty syndrome'[Title/Abstract] |
|  | 8 | 'Geriatric assessment'[MeSH Terms] OR 'Geriatric assessment'[Title/Abstract] |
|  | 9 | 'Grip strength'[Title/Abstract] |
|  | 10 | #1 OR #2 OR #3 OR #4 OR #5 OR #6 OR #7 OR #8 OR #9 |
|  | 11 | 'Cognitive impairment'[Title/Abstract] OR 'Cognitive impairment'[MeSH Terms] |
|  | 12 | 'Cognitive dysfunction'[Title/Abstract] |
|  | 13 | 'Cogniti* impairment'[Title/Abstract] |
|  | 14 | 'Cogniti* dysfunction'[Title/Abstract] |
|  | 15 | 'Alzheimer'[MeSH Terms] OR Alzheimer[Title/Abstract] |
|  | 16 | Dementia[Title/Abstract] OR Dementia[MeSH Terms] |
|  | 17 | 'Dement*'[Title/Abstract] |
|  | 18 | Cognition[Title/Abstract] |
|  | 19 | 'Mental Deterioration'[Title/Abstract] |
|  | 20 | #11 OR #12 OR #13 OR #14 OR #15 OR #16 OR #17 OR #18 OR #19 |
|  | 21 | 'Chronic kidney disease'[Title/Abstract] OR 'Chronic kidney disease'[MeSH Terms] |
|  | 22 | 'Chronic kidney*'[Title/Abstract] |
|  | 23 | 'Chronic renal*'[Title/Abstract] |
|  | 24 | 'CKD'[Title/Abstract] OR 'ESRD'[Title/Abstract] OR 'ESKD'[Title/Abstract] |
|  | 25 | 'End-stage kidney disease'[Title/Abstract] OR 'End-stage kidney disease'[MeSH Terms] |
|  | 26 | 'End-stage renal disease'[Title/Abstract] |
|  | 27 | Dialysis[Title/Abstract] |
|  | 28 | 'Peritoneal dialysis'[MeSH Terms] OR 'Peritoneal dialysis'[Title/Abstract] |
|  | 29 | Hemodialysis[Title/Abstract] OR Hemodialysis[MeSH Terms] |
|  | 30 | 'Pre-dialy*'[Title/Abstract] |
|  | 31 | 'Dialy*'[Title/Abstract] |
|  | 32 | #21 OR #22 OR #23 OR #24 OR #25 OR #26 OR #27 OR #28 OR #29 OR #30 OR #31 |
|  | 33 | #10 AND #20 AND #32 |
|  |  | 313 Search Results 27/02/2026) |
| 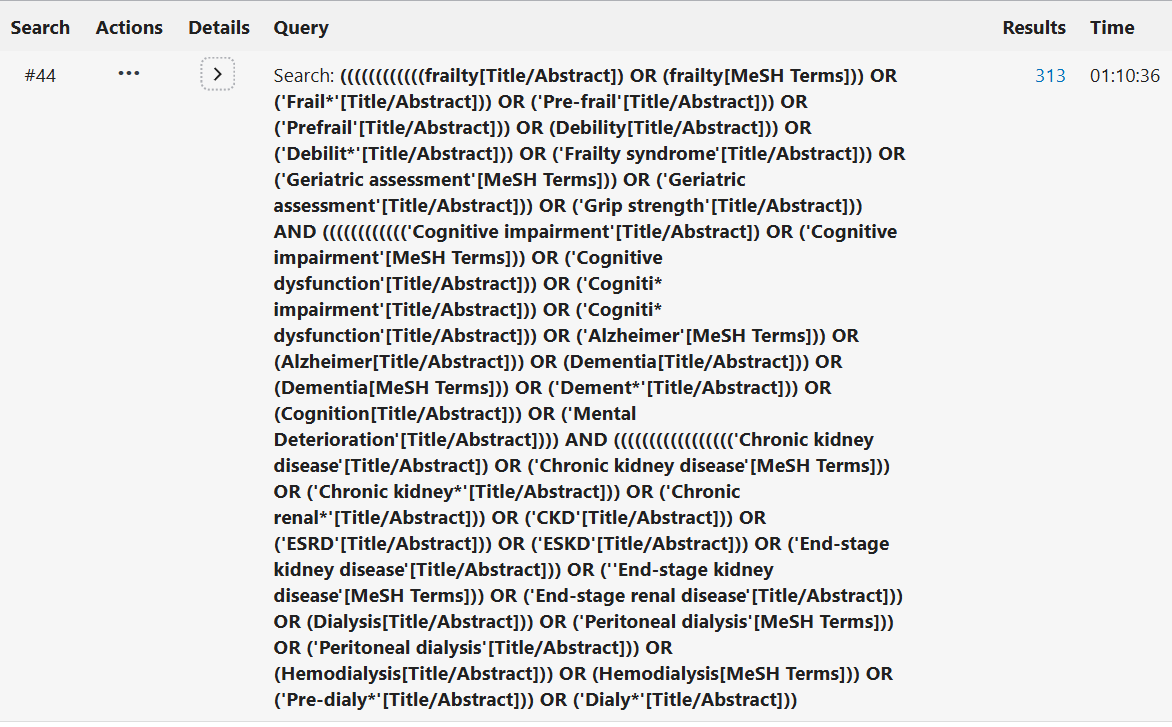 | | |
| **2.Embase**  **(n=1810)** | 1 | 'frailty'/exp OR 'frailty':ti,ab,kw |
|  | 2 | 'frail*':ti,ab,kw |
|  | 3 | ‘prefrailty'/exp OR ‘prefrailty':ti,ab,kw |
|  | 4 | 'frailty syndrome'/exp OR 'frailty syndrome':ti,ab,kw |
|  | 5 | debility:ti,ab,kw |
|  | 6 | 'debilit*':ti,ab,kw |
|  | 7 | 'geriatric assessment'/exp OR 'geriatric assessment':ti,ab,kw |
|  | 8 | 'grip strength'/exp OR 'grip strength':ti,ab,kw |
|  | 9 | #1 OR #2 OR #3 OR #4 OR #5 OR #6 OR #7 OR #8 |
|  | 10 | 'cognitive defect'/exp OR 'cognitive defect':ti,ab,kw OR 'cognitive impairment':ti,ab,kw |
|  | 11 | 'cogniti* dysfunction':ti,ab,kw |
|  | 12 | 'cogniti* impairment':ti,ab,kw |
|  | 13 | 'alzheimer disease'/exp OR 'alzheimer disease':ti,ab,kw |
|  | 14 | 'dementia'/exp OR 'dementia':ti,ab,kw |
|  | 15 | 'dement*':ti,ab,kw |
|  | 16 | 'cognition'/exp OR 'cognition':ti,ab,kw |
|  | 17 | 'mental deterioration'/exp OR 'mental deterioration':ti,ab,kw |
|  | 18 | #10 OR #11 OR #12 OR #13 OR #14 OR #15 OR #16 OR #17 |
|  | 19 | 'chronic kidney failure'/exp OR 'chronic kidney failure':ti,ab,kw OR 'chronic kidney disease':ti,ab,kw |
|  | 20 | 'chronic kidney*':ti,ab,kw |
|  | 21 | 'chronic renal*':ti,ab,kw |
|  | 22 | 'end stage renal disease'/exp OR 'end stage renal disease':ti,ab,kw |
|  | 23 | 'hemodialysis'/exp OR 'hemodialysis':ti,ab,kw |
|  | 24 | 'peritoneal dialysis'/exp OR 'peritoneal dialysis':ti,ab,kw |
|  | 25 | 'pre dialy*':ti,ab,kw |
|  | 26 | ‘C’:ti,ab,kw |
|  | 27 | #19 OR #20 OR #21 OR #22 OR #23 OR #24 OR #25 OR #26 |
|  | 28 | #9 AND #18 AND #27 |
|  |  | 1810 Search Results (27/02/2026) |
| 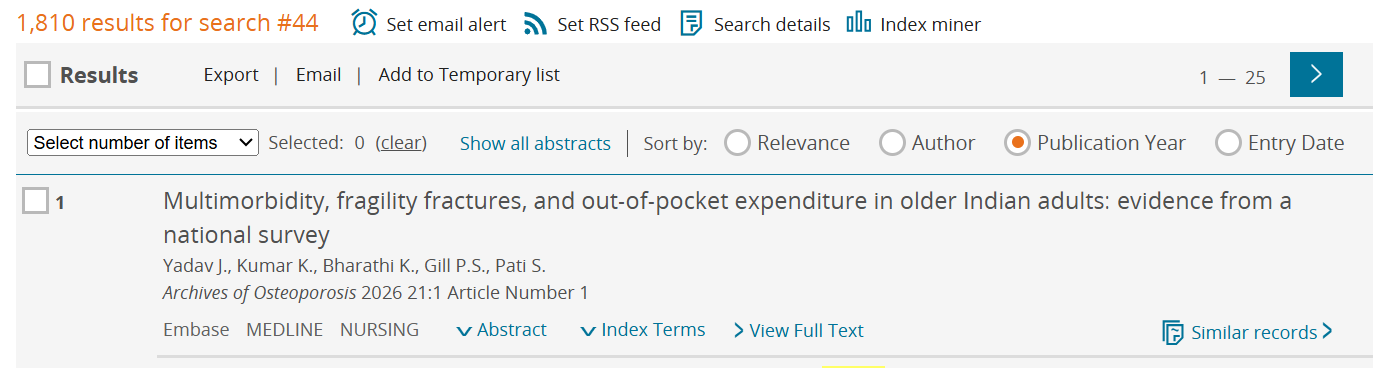 | | |
| **3.CINAHL**  **(n=183)** | 1 | "frailty" OR (MH "Frailty Syndrome") |
|  | 2 | ‘frail*’ |
|  | 3 | ‘pre-frail’ |
|  | 4 | ‘debilit*’ |
|  | 5 | debility |
|  | 6 | (MH "Geriatric Assessment+") OR ‘Geriatric Assessment’ |
|  | 7 | (MH "Grip Strength") OR ‘Grip Strength’ |
|  | 8 | #1 OR #2 OR #3 OR #4 OR #5 OR #6 OR #7 |
|  | 9 | (MH "Cognition") OR (MH "Cognition Disorders") OR "cognitive dysfunction" |
|  | 10 | (MH "Mild Cognitive Impairment") OR "cognitive impairment" |
|  | 11 | ‘cogniti* dysfunction’ |
|  | 12 | ‘cogniti* impairment’ |
|  | 13 | (MH "Alzheimer's Disease") OR "Alzheimer" |
|  | 14 | (MH "Dementia") |
|  | 15 | ‘dement*’ |
|  | 16 | "mental deterioration" |
|  | 17 | #9 OR #10 OR #11 OR #12 OR #13 OR #14 OR #15 OR #16 |
|  | 18 | (MH "Kidney Failure, Chronic") OR (MH "Renal Insufficiency, Chronic") OR "chronic kidney disease" |
|  | 19 | ‘chronic kidney*’ |
|  | 20 | ‘chronic renal*’ |
|  | 21 | ‘CKD’ |
|  | 22 | ‘ESRD’ |
|  | 23 | ‘ESKD’ |
|  | 24 | 'End-stage kidney disease' OR 'End-stage renal disease' OR 'End stage renal disease' OR 'End stage kidney disease' |
|  | 25 | (MH "Dialysis") |
|  | 26 | (MH "Peritoneal Dialysis") OR (MH "Peritoneal Dialysis, Continuous Ambulatory") |
|  | 27 | (MH "Hemodialysis") OR (MH "Continuous Venovenous Hemodialysis") OR (MH "Continuous Arteriovenous Hemodialysis") |
|  | 28 | ‘pre-dialy*’ |
|  | 29 | ‘dialy*’ |
|  | 30 | #18 OR #19 OR #20 OR #21 OR #22 OR #23 OR #24 OR #25 OR #26 OR #27 OR #28 OR #29 |
|  | 31 | #8 AND #17 AND #30 |
|  |  | 183 Search Results (27/02/2026) |
| 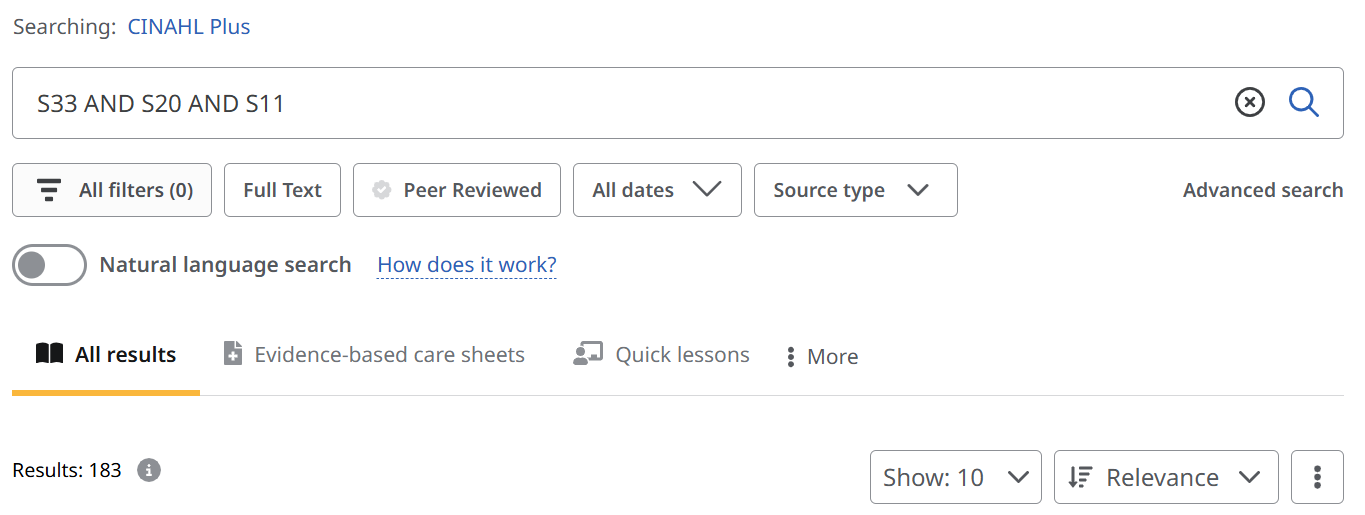 | | |
| **4. Web of Science**  **(n=422)** | 1 | frailty (All Fields) |
|  | 2 | 'frail*' (All Fields) |
|  | 3 | 'pre-frail' (All Fields) OR prefrail (All Fields) |
|  | 4 | debility (All Fields) OR 'debilit*' (All Fields) |
|  | 5 | 'frailty syndrome' (All Fields) |
|  | 6 | 'geriatric assessment' (All Fields) |
|  | 7 | 'grip strength' (All Fields) |
|  | 8 | #1 OR #2 OR #3 OR #4 OR #5 OR #6 OR #7 |
|  | 9 | 'cognitive impairment' (All Fields) |
|  | 10 | 'cognitive dysfunction' (All Fields) |
|  | 11 | 'cogniti* dysfunction' (All Fields) |
|  | 12 | 'cogniti* impairment' (All Fields) |
|  | 13 | Alzheimer (All Fields)Alzheimer |
|  | 14 | dementia (All Fields) |
|  | 15 | 'dement*' (All Fields) |
|  | 16 | cognition (All Fields) |
|  | 17 | 'mental deterioration' (All Fields) |
|  | 18 | #9 OR #10 OR #11 OR #12 OR #13 OR #14 OR #15 OR #16 OR #17 |
|  | 19 | 'chronic kidney disease' (All Fields) |
|  | 20 | 'chronic kidney*' (All Fields) |
|  | 21 | 'chronic renal*' (All Fields) |
|  | 22 | 'CKD' (All Fields) |
|  | 23 | 'ESRD' (All Fields) |
|  | 24 | 'ESKD' (All Fields) |
|  | 25 | 'endstage kidney disease' (All Fields) OR  'endstage kidney disease' (All Fields) OR 'end-stage renal disease' (All Fields) OR 'endstage renal disease' (All Fields) |
|  | 26 | 'pre-dialy*' (All Fields) |
|  | 27 | 'predialy*' (All Fields) |
|  | 28 | #19 OR #20 OR #21 OR #22 OR #23 OR #24 OR #25 OR #26 OR #27 |
|  | 29 | #8 AND #18 AND #27 |
|  |  | 422 Search Results (27/02/2026) |
| 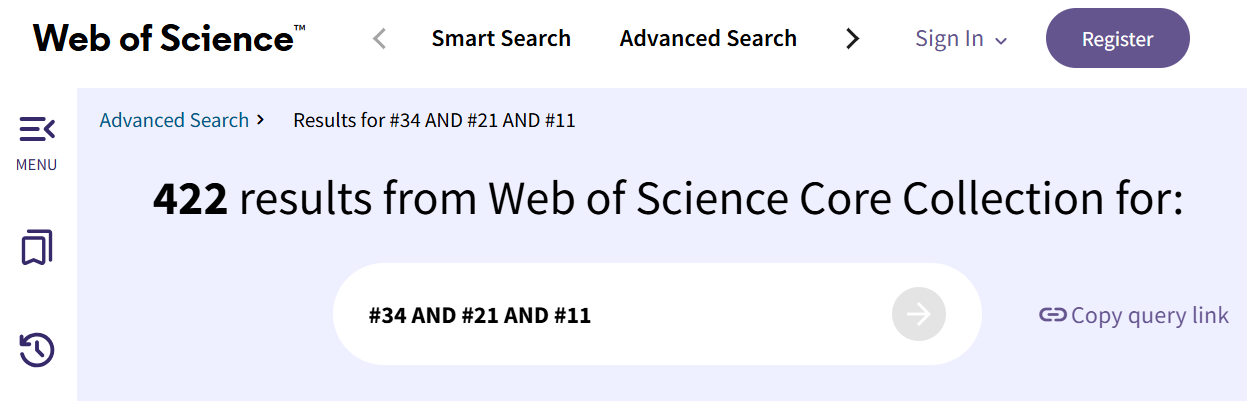 | | |
| **5. Scopus**  **(n=1354)** | 1 | TITLE-ABS-KEY ( frailty ) |
|  | 2 | TITLE-ABS-KEY ( 'frail*' ) |
|  | 3 | TITLE-ABS-KEY ( 'pre-frail' ) OR  TITLE-ABS-KEY ( prefrail ) |
|  | 4 | TITLE-ABS-KEY ( debility ) OR TITLE-ABS-KEY ( 'debilit*' ) |
|  | 5 | TITLE-ABS-KEY ( 'frailty syndrome' ) |
|  | 6 | TITLE-ABS-KEY ( 'geriatric assessment' ) |
|  | 7 | TITLE-ABS-KEY ( 'grip strength' ) |
|  | 8 | #1 OR #2 OR #3 OR #4 OR #5 OR #6 OR #7 |
|  | 9 | TITLE-ABS-KEY ( 'cognitive impairment' ) |
|  | 10 | TITLE-ABS-KEY ( 'cognitive dysfunction' ) |
|  | 11 | TITLE-ABS-KEY ( 'cogniti* dysfunction' ) |
|  | 12 | TITLE-ABS-KEY ( 'cogniti* impairment' ) |
|  | 13 | TITLE-ABS-KEY ( Alzheimer ) |
|  | 14 | TITLE-ABS-KEY ( dementia ) |
|  | 15 | TITLE-ABS-KEY ( 'dement*' ) |
|  | 16 | TITLE-ABS-KEY ( cognition ) |
|  | 17 | TITLE-ABS-KEY ( 'mental deterioration' ) |
|  | 18 | #9 OR #10 OR #11 OR #12 OR #13 OR #14 OR #15 OR #16 OR #17 |
|  | 19 | TITLE-ABS-KEY ( 'chronic kidney disease' ) |
|  | 20 | TITLE-ABS-KEY ( 'chronic kidney*' ) |
|  | 21 | ITLE-ABS-KEY ( 'chronic renal*' ) |
|  | 22 | TITLE-ABS-KEY ( 'CKD' ) |
|  | 23 | TITLE-ABS-KEY ( 'ESRD' ) |
|  | 24 | TITLE-ABS-KEY ( 'ESKD' ) |
|  | 25 | TITLE-ABS-KEY ( 'end-stage kidney disease' ) OR TITLE-ABS-KEY ( 'endstage kidney disease' ) OR TITLE-ABS-KEY ( 'end-stage renal disease' ) OR TITLE-ABS-KEY ( 'endstage renal disease' ) |
|  | 26 | TITLE-ABS-KEY ( 'pre-dialy*' ) |
|  | 27 | TITLE-ABS-KEY ( 'pre-dialy*' ) |
|  | 28 | #19 OR #20 OR #21 OR #22 OR #23 OR #24 OR #25 OR #26 OR #27 |
|  | 29 | #8 AND #18 AND #27 |
|  |  | 1354 Search Results (27/02/2026) |
| 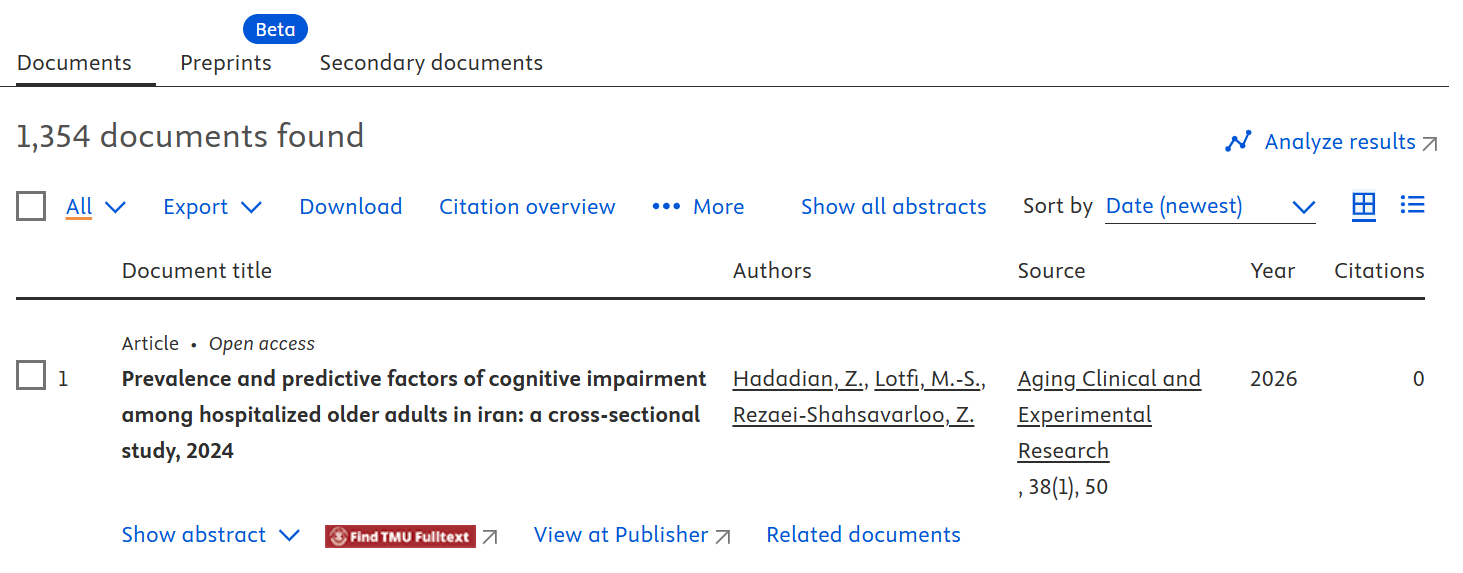 | | |
| **6. Cochrane**  **(n=64)** | 1 | MeSH descriptor: [Frailty] explode all trees |
|  | 2 | ('frail*'):ti,ab,kw |
|  | 3 | ('pre-frail'):ti,ab,kw |
|  | 4 | ('prefrail'):ti,ab,kw |
|  | 5 | ('debility'):ti,ab,kw |
|  | 6 | ('debilit*'):ti,ab,kw |
|  | 7 | ('frailty syndrome'):ti,ab,kw |
|  | 8 | MeSH descriptor: [Geriatric Assessment] explode all trees |
|  | 9 | MeSH descriptor: [Hand Strength] explode all trees |
|  | 10 | #1 OR #2 OR #3 OR #4 OR #5 OR #6 #7 OR #8 OR #9 |
|  | 11 | MeSH descriptor: [Cognitive Dysfunction] explode all trees |
|  | 12 | ('cognitive impairment'):ti,ab,kw |
|  | 13 | ('cogniti* dysfunction'):ti,ab,kw |
|  | 14 | ('cogniti* impairment'):ti,ab,kw |
|  | 15 | MeSH descriptor: [Alzheimer Disease] explode all trees |
|  | 16 | MeSH descriptor: [Dementia] explode all trees |
|  | 17 | ('dement*'):ti,ab,kw |
|  | 18 | MeSH descriptor: [Cognition] explode all trees |
|  | 19 | ('mental deterioration'):ti,ab,kw |
|  | 20 | #11 #12 #13 #14 #15 #16 OR #17 OR #18 OR #19 |
|  | 21 | MeSH descriptor: [Renal Insufficiency, Chronic] explode all trees |
|  | 22 | ('chronic kidney disease'):ti,ab,kw |
|  | 23 | ('chronic kidney*'):ti,ab,kw |
|  | 24 | ('chronic renal*'):ti,ab,kw |
|  | 25 | MeSH descriptor: [Renal Insufficiency] explode all trees |
|  | 26 | ('CKD'):ti,ab,kw |
|  | 27 | ('ESRD'):ti,ab,kw |
|  | 28 | ('ESKD'):ti,ab,kw |
|  | 29 | MeSH descriptor: [Kidney Failure, Chronic] explode all trees |
|  | 30 | ('Endstage kidney disease'):ti,ab,kw |
|  | 31 | ('End-stage kidney disease'):ti,ab,kw |
|  | 32 | ('End-stage renal disease'):ti,ab,kw |
|  | 33 | ('End stage renal disease'):ti,ab,kw |
|  | 34 | MeSH descriptor: [Dialysis] explode all trees |
|  | 35 | MeSH descriptor: [Peritoneal Dialysis] explode all trees |
|  | 36 | MeSH descriptor: [Renal Dialysis] explode all trees |
|  | 37 | ('pre-dialy*'):ti,ab,kw |
|  | 38 | ('predialy*'):ti,ab,kw |
|  | 39 | ('dialy*'):ti,ab,kw |
|  | 40 | #21 OR #23 OR #24 OR #25 OR #26 OR #27 OR #28 OR #29 OR #30 OR #31 OR #32 OR #33 OR #34 OR #35 OR #36 OR #37 OR #38 OR #39 |
|  | 41 | #10 AND #20 AND #40 |
|  | 42 | 64 Search Results (27/02/2026) |
| 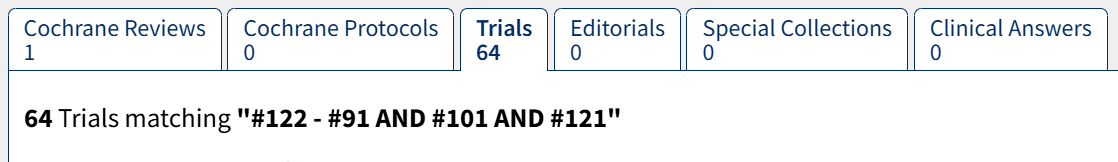 | | |

Supplementary Table S2. A list of excluded studies after a full-text review

| **No.** | **Article** | | **Reason for exclusion** |
| --- | --- | --- | --- |
| 1 | Ambulatory blood pressure patterns, cognitive function, and frailty in CKD: Chronic renal insufficiency cohort (CRIC) study. | | Conference Abstract |
| 2 | Assessing the Quality of Life in Older Patients with Endstage Kidney Disease Receiving Palliative Care. | | Conference Abstract |
| 3 | Prevalence of Frailty and Symptom Burden in Patients on Staff-Assisted Peritoneal Dialysis. | | Conference Abstract |
| 4 | The Prevalence of Cognitive Impairment in Chinese Peritoneal Dialysis Patients. | | Conference Abstract |
| 5 | A practical solution to screen cognitive frailty among hemodialysis patients using a game-based intradialytic exercise with wearable sensors. | | Conference Abstract |
| 6 | Prevalence of frailty and its association with quality of life, clinical and biochemical markers in end-stage renal disease patients under dialysis. | | Conference Abstract |
| 7 | Frail phenotype versus EGFR in predicting longitudinal outcomes for elderly with chronic kidney disease. | | Conference Abstract |
| 8 | Lower Functional Status and Perceived Health Status Are Associated With Poorer Quality of Life in Older People With ESKD. | | Conference Abstract |
| 9 | PD assistance and relationship to co-existing geriatric syndromes in incident peritoneal dialysis therapy patients. | | Did not report the association between frailty and cognitive impairment. |
| 10 | Association of 24-hour ambulatory blood pressure patterns with cognitive function and physical functioning in CKD. | | Did not report the association between frailty and cognitive impairment. |
| 11 | Anxiety, depression and post-traumatic stress disorder in patients on hemodialysis in the setting of the pandemic, inflation, and the Beirut blast: a cross-sectional study. | | Did not report the association between frailty and cognitive impairment. |
| 12 | Association of Urine Biomarkers of Kidney Tubule Injury and Dysfunction With Frailty Index and Cognitive Function in Persons With CKD in SPRINT. | | Did not report the association between frailty and cognitive impairment. |
| 13 | Geriatric Assessment and the Relation with Mortality and Hospitalizations in Older Patients Starting Dialysis. | | Did not report the association between frailty and cognitive impairment. |
| 14 | Limited health literacy and adverse outcomes among kidney transplant candidates. | | Did not report the association between frailty and cognitive impairment. |
| 15 | The Impact of Frailty and Severe Cognitive Impairment on Survival Time and Time to Initiate Dialysis in Older Adults With Advanced Chronic Kidney Disease: A Prospective Observational Cohort Study. | | Did not report the association between frailty and cognitive impairment. |
| 16 | Frailty, physical function and affective status in elderly patients on hemodialysis. | | Could not calculate OR to asess the association between frailty and cognitive impairment. |
| 17 | Prevalence and Prognosis of Coexisting Frailty and Cognitive Impairment in Patients on Continuous Ambulatory Peritoneal Dialysis. | | Could not calculate OR to asess the association between frailty and cognitive impairment. |
| 18 | Frailty, Health-Related Quality of Life, Cognition, Depression, Vitamin D and Health-Care Utilization in an Ambulatory Adult Population With Type 1 or Type 2 Diabetes Mellitus and Chronic Kidney Disease: A Cross-Sectional Analysis. | | Could not calculate OR to asess the association between frailty and cognitive impairment. |
| 19 | Frailty and chronic kidney disease: associations and implications. | | Could not calculate OR to asess the association between frailty and cognitive impairment. |
| 20 | Association of Frailty With Nutritional Status in Patients With Chronic Kidney Disease. | | Could not calculate OR to asess the association between frailty and cognitive impairment. |
| 21 | Subclinical cognitive impairment in chronic kidney disease is associated with frailty and reduced quality of life. | | Could not calculate OR to asess the association between frailty and cognitive impairment. |
| 22 | The Prevalence of Frailty and its Association with Cognitive Dysfunction among Elderly Patients on Maintenance Hemodialysis: A Cross-Sectional Study from South India. | | Could not calculate OR to asess the association between frailty and cognitive impairment. |
| 23 | The prevalence of frailty according to kidney function and its association with cognitive impairment, nutritional status, and clinical outcome | Could not calculate OR to assess the association between frailty and cognitive impairment | |

Supplementary Table S3. Measurements and Cutoff Values of Cognitive Impairment and Frailty in Included Studies (n=17)

| **Study name** | **Frailty measurement** | **Cut-off value of frailty** | **Cognitive impairment measurement** | **Cut-off value of cognitive impairment** | **Covariates adjusted** |
| --- | --- | --- | --- | --- | --- |
| Anderson et al. (2023) | Fried Frailty Phenotype, Frailty Index, Edmonton Frailty Scale, and Clinical Frailty Scale | N/I | MoCA | MoCA <26 | Dementia, parathyroid hormone, hemodialysis vintage, education level, diabetes, hemoglobin, albumin, smoking status, patient health questionnaire - 9, previous cerebral-vascular accident |
| Chu et al. (2021) | Fried Frailty Phenotype | - Frail: ≥ 3 | 3MS | 3MS<80 | Age group, sex, race, education level of high school or below, dialysis type (no dialysis, hemodialysis, or peritoneal dialysis), diabetes status and donor type (deceased donor compared to living donor) |
| Erken & Erken (2023) | Clinical Frailty Index | - Good condition:1–3  - Susceptible to frailty: 4  - Frail: 5–7 | MoCA | MoCA ≤ 25 | N/I |
| Erken et al. (2019) | Clinical frailty index | - Frail: ≥ 4 | MoCA | MoCA <24 | N/I |
| Franco et al. (2023) | Fried Frailty Phenotype | - Frail: ≥ 3 | MoCA | MoCA <24 | N/I |
| Gesualdo et al. (2020) | Fried Frailty Phenotype | - Non-frail: 0  - Pre-frail:1-2  - Frail: ≥ 3 | ACE-R | ACE-R<78 | N/I |
| Guo et al. (2022) | Fried Frailty Phenotype | - Non-frail: 0  - Pre-frail:1-2  - Frail: ≥ 3 | MoCA | MoCA <26 | N/I |
| Hong et al. (2024) | Fried Frailty Phenotype | - Frail: ≥ 3 | Objective measures | N/I | N/I |
| Jafari et al. (2020) | Fried Frailty Phenotype | - Non-frail: 0  - Pre-frail:1-2  - Frail: ≥ 3 | MoCA | MoCA ≤ 24 | N/I |
| Lee et al. (2021) | A modified FRAIL scale | - Pre-frailty: 1 or 2 components  - Frailty: more than 2 components | A validated algorithm | N/I | N/I |
| McAdams-DeMarco et al. (2015) | Fried Frailty Phenotype | - Non- Frail: 0-1  - Intermediate frail: 2  - Frail: ≥ 3 | 3MS | 3MS <80 | N/I |
| Nixon et al. (2020) | Fried Frailty Phenotype | - Frail: ≥ 3 | MMSE | MMSE ≤27 | N/I |
| Novais et al. (2021) | Fried Frailty Phenotype | - Non-frail: 0  - Pre-frail:1-2  - Frail: ≥ 3 | MMSE | MMSE ≤ 25 | N/I |
| Poveda et al. (2016) | FRAIL questionnaire | - Non-frail: 0  - Pre-frail:1-2  - Frail: ≥ 3 | MMSE | N/I | N/I |
| S. Vettoretti et al. (2020) | Fried Frailty Phenotype | - Frail: ≥ 3 | MMSE | MMSE ≤ 23 | N/I |
| Thind et al. (2022) | Edmonton Frail Scale | - Frail ≥8  - Vulnerable: 6–7  - Not frail <6 | MoCA | MoCA <26 | N/I |
| Chen et al., (2025) | Fried Frailty Phenotype | - Non-frail: 0  - Pre-frail:1-2  - Frail: ≥ 3 | MMSE | > 17 for illiterate individuals, > 20 for those with primary education, and > 24 for individuals with junior high school education or above. | N/I |

Abbreviations: 3MS: Modified Mini-Mental State test; FRAIL: fatigue, resistance, aerobic capacity, and loss of weight; MoCA: Montreal Cognitive Assessment; MMSE: Mini-Mental State Examination; ACE-R: Addenbrooke’s Cognitive Examination-Revised; N/I: No information.

Supplementary Table S4. Quality Assessment of Included Studies (n = 17)

**(Newcastle-Ottawa for Cohort Studies and Adapted Newcastle-Ottawa Scale for Cross-Sectional Studies)**

| **Cross-Sectional** | | | | | | | | | | | | | |
| --- | --- | --- | --- | --- | --- | --- | --- | --- | --- | --- | --- | --- | --- |
| Study | Selection  (max 3 stars) | | | | Comparability  (max 2 stars) | | Outcome  (max 3 stars) | | | Total Score  (max 8) | | Evaluation | |
|  | Representativeness of Sample | Sample Size | Non-included subjects | Subtotal | Based on Design and Analysis | Subtotal | Assessment of Outcome | Statistical Test | Subtotal |  |  |  |  |
| S. Vettoretti et al. (2020) | - | - | 🟊 | 1 | - | 0 | 🟊🟊 | 🟊 | 3 | 4 | | High risk | |
| Poveda et al. (2016) | - | - | 🟊 | 1 | 🟊🟊 | 2 | 🟊🟊 | 🟊 | 3 | 6 | | Medium risk | |
| Gesualdo et al. (2020) | - | - | 🟊 | 1 | 🟊🟊 | 2 | 🟊🟊 | 🟊 | 3 | 6 | | Medium risk | |
| Erken et al. (2019) | - | - | 🟊 | 1 | 🟊🟊 | 2 | 🟊🟊 | 🟊 | 3 | 6 | | Medium risk | |
| Hong et al. (2024) | 🟊 | 🟊 | 🟊 | 3 | 🟊🟊 | 2 | 🟊🟊 | 🟊 | 3 | 8 | | Low risk | |
| Erken & Erken (2023) | - | - | 🟊 | 1 | 🟊🟊 | 2 | 🟊🟊 | 🟊 | 3 | 6 | | Medium risk | |
| Novais et al. (2021) | - | - | - | 0 | 🟊🟊 | 2 | 🟊🟊 | 🟊 | 3 | 5 | | High risk | |
| Nixon et al. (2020) | - | - | 🟊 | 1 | 🟊🟊 | 2 | 🟊🟊 | 🟊 | 3 | 6 | | Medium risk | |
| Chen et al., (2025) | - | 🟊 | 🟊 | 2 | 🟊🟊 | 2 | 🟊🟊 | 🟊 | 3 | 7 | | Low risk | |
| **Cohort** | | | | | | | | | | | | | |
| Study | Selection  (max 4 stars) | | | | | Comparability  (max 2 stars) | | Outcome  (max3 stars) | | | | Total Score  (max 9) | Evaluation |
|  | Representative of the exposed cohort | Selection of the non-exposed cohort | Ascertainment of exposure | Demonstration of outcome | Subtotal | Based on Design and Analysis | Subtotal | Assessment of outcome | Follow up | Adequacy of follow up | Sub total |  |  |
| McAdam- DeMarco et at. (2015) | 🟊 | 🟊 | 🟊 | 🟊 | 4 | 🟊🟊 | 2 | 🟊 | 🟊 | 🟊 | 3 | 9 | Low risk |
| Anderson et al. (2023) | 🟊 | 🟊 | 🟊 | 🟊 | 4 | 🟊🟊 | 2 | 🟊 | 🟊 | 🟊 | 3 | 9 | Low risk |
| Guo et al. (2022) | 🟊 | 🟊 | - | 🟊 | 3 | 🟊🟊 | 2 | 🟊 | 🟊 | 🟊 | 3 | 8 | Low risk |
| Jafari et al. (2020) | - | 🟊 | 🟊 | 🟊 | 3 | - | 0 | 🟊 | 🟊 | 🟊 | 3 | 6 | Medium risk |
| Chu et al. (2021) | 🟊 | 🟊 | - | 🟊 | 3 | 🟊🟊 | 2 | 🟊 | 🟊 | - | 2 | 7 | Medium risk |
| Franco et al. (2023) | - | 🟊 | 🟊 | 🟊 | 3 | 🟊🟊 | 2 | 🟊 | 🟊 | 🟊 | 3 | 8 | Low risk |
| Thind et al. (2022) | - | 🟊 | 🟊 | 🟊 | 3 | - | 0 | 🟊 | 🟊 | - | 5 | 8 | High risk |
| Lee et al. (2021) | - | 🟊 | 🟊 | 🟊 | 3 | 🟊🟊 | 2 | 🟊 | 🟊 | - | 2 | 8 | Low risk |

Supplementary Table S5. Sensitivity analysis

| **Sensitivity analysis** | **No of studies** | **I^2^** | **OR** | **95% CI** | **p** |
| --- | --- | --- | --- | --- | --- |
| Excluded the study with the largest OR | 16 | 94.07 | 2.84 | 1.73 – 4.68 | <0.001 |
| Excluded the studies using the MMSE | 12 | 95.79 | 3.26 | 1.78- 5.98 | <0.001 |

Abbreviations: MMSE: Mini-Mental State Examination
